# New Targeted Agents in Myasthenia Gravis and Future Therapeutic Strategies

**DOI:** 10.3390/jcm11216394

**Published:** 2022-10-28

**Authors:** Daniel Sánchez-Tejerina, Javier Sotoca, Arnau Llaurado, Veronica López-Diego, Raul Juntas-Morales, Maria Salvado

**Affiliations:** Clinic of Neuromuscular Disorders and Rare Diseases, Neurology Department, Hospital Universitari Vall d’Hebron, Vall d’Hebron Research Institute, European Reference Network for Neuromuscular and Rare Diseases EURO-NMD, 08035 Barcelona, Spain

**Keywords:** myasthenia gravis, complement inhibitors, B-cell, monoclonal antibody, FcRn inhibitors, targeted treatments

## Abstract

Myasthenia gravis (MG) is a chronic autoimmune disease for which multiple immunomodulatory therapies are available. Nevertheless, MG has a significant impact on patient quality of life. In recent years, experts’ main efforts have focused on optimizing treatment strategies, since disease burden is considerably affected by their safety and tolerability profiles, especially in patients with refractory phenotypes. This article aims to offer neurologists caring for MG patients an overview of the most innovative targeted drugs specifically designed for this disease and summarizes the recent literature and more recent evidence on agents targeting B cells and plasmablasts, complement inhibitors, and neonatal fragment crystallizable receptor (FcRn) antagonists. Positive clinical trial results have been reported, and other studies are ongoing. Finally, we briefly discuss how the introduction of these novel targeted immunological therapies in a changing management paradigm would affect not only clinical outcomes, disease burden, safety, and tolerability, but also health spending in a condition that is increasingly managed based on a patient-centred model.

## 1. Introduction

Myasthenia gravis (MG) is a chronic autoimmune disease in which an extensive range of immunomodulatory therapies have conventionally been used to achieve clinical remission or, at least, minimal manifestation status according to the classification of the Myasthenia Gravis Foundation of America [1].

Bearing in mind the individual clinical, serological, and thymic phenotype of the patient, the most recent international consensus guidelines on the management of MG provide updated recommendations for optimal treatment based on the patient’s situation and comorbidities [2].

As a chronic disabling condition with a commonly fluctuating course, MG usually entails unpredictable hospitalizations, difficulties reconciling work and home life, and, consequently, psychological or psychiatric disorders and/or sleep disturbances. Consequently, MG has an inevitable negative impact on the patient’s quality of life [3,4], and treatment-related adverse effects and tolerability problems contribute to the notable disease burden reported [5].

In recent years, interest has been growing in the emotional, socio-familial, and humanistic aspects of MG, as well as the effects of medical interventions on disease burden [6,7,8,9,10]. This scenario is especially crucial for patients with treatment-refractory MG [11,12].

Hence, while traditional treatment regimens are based on relatively non-specific pharmacologic strategies (usually pyridostigmine alone or in combination with corticosteroids or non-steroidal immunosuppressants) with a problematic tolerance and safety profile, novel drug alternatives are urgently required. In that sense, a focus on each of the factors involved in the disease background and an understanding of their role in pathophysiology are helping investigators to develop new specific targeted drugs.

Novel drugs for MG include molecules targeting B cells, plasmablasts, complement inhibitors, and neonatal fragment crystallizable receptor (FcRn) antagonists. In this review, our aims were to analyze the recent literature and evidence on these innovative therapies and to briefly discuss the impact of therapy on clinical outcomes, acute exacerbations, adverse events, tolerability, and quality of life.

## 2. Pathophysiology of Myasthenia Gravis

MG is caused by the failure of neuromuscular transmission resulting from the binding of autoantibodies to signaling proteins—mostly the nicotinic acetylcholine receptor (AChR)—at the neuromuscular junction (NMJ). The pathophysiology of MG is extremely complex and multifactorial, involving interlinked environmental, genetic, and epigenetic factors, which are responsible for the loss of immune tolerance [13].

Presumably, antigen-presenting cells promote AChR-antibody (AChR)–mediated responses by enhancing CD4+ T-cell activation through HLA, triggering upregulation of IL-4 and IL-6 and, consequently, B-cell stimulation and AChR-ab production. These mechanisms then probably lead to the activation of mature T and B lymphocytes in the thymus. Activated T cells produce pro-inflammatory cytokines, such as IFN-γ and IL-17, leading to an imbalance between deficient regulatory T cells (Treg) and hyperactivated Th17 cells, which further enhances antibody production [14,15,16,17].

In patients with thymoma, some authors were able to prove a deficiency of indispensable molecular components for immune tolerance, as those provided by the AIRE gene [18].

As mentioned above, the autoantibody-mediated response against signaling proteins at the NMJ plays an important role in pathophysiology. AChR-abs are detected in about 80–85% of MG patients. Most seronegative patients have antibodies against muscle-specific kinase (MuSK) (6% of generalized MG [gMG]) or anti-LrP4 antibodies (low-density lipoprotein receptor type 4) (2% of gMG) [19]. Yet, about 15% of MG patients, from various types of populations, remain seronegative [20].

Although we are not able to detect antibodies in seronegative MG, there is some evidence for complementary activation by IgG1 antibodies against clustered AChR, as complement deposits have been identified in biopsy specimens of thymus tissue [21,22] and intercostal muscle [23].

AChR-abs are mainly generated by long-lived plasma cells. The most direct and intuitive mechanism of action in AChR-MG is the direct antibody blockade of the AChR [24,25,26]. In these patients, the main response mechanisms belong to subclasses IgG1, IgG2, and IgG3, which induce the internalization and degradation of AChR by crosslinking the receptors in the postsynaptic region. This response triggers an important effector mechanism through a cascade of immune reactions, including complement activation (which leads to destruction of neuromuscular endplate structures by the membrane attack complex), reduction in AChRs in the membrane, loss of postsynaptic folding, and an increase in intersynaptic distance (which leads to malfunction of neuromuscular transmission) [20].

MuSK is a key molecule with respect to AChR clustering. MuSK antibodies are thought to be produced by short-lived plasmablasts. They are mainly IgG4 and do not trigger antibody-mediated complement-fixing endplate destruction. MuSK antibodies disrupt the clustering of AChR, thus hampering the interaction between LRP4 and MuSK (by blocking protein-protein interaction), inducing the dispersion of preformed agrin-independent AChR clusters, and disturbing the molecular structure underlying the endplate region [27,28,29].

LRP4 antibodies belong to the complement-activating IgG1 and IgG3 subclasses and can disrupt agrin-LRP4 signaling in the postsynaptic membrane [30]. These antibodies are not definitory of MG diagnosis, as their role remains unclear. Other muscle antibodies that can be detected in some MG patients include antibodies against agrin, cortactin, collQ, acetylcholinesterase (AChE), Kv1.4, titin, and ryanodine receptor, although their clinical implications have yet to be established [24,25,26].

## 3. New Therapeutic Strategies in Myasthenia Gravis

### 3.1. B-Cell Inhibitors

Under the influence of helper T cells and certain cytokines, B cells differentiate into memory B cells, plasmablasts, and plasma cells in the thymic germinal centers. One of the fundamental functions of plasmablasts and plasma cells is that of secreting antibodies, including pathogenic antibodies. Consequently, they could be considered the main effector cells in the pathogenesis of MG. Plasma cell populations have been classified according to the molecules expressed on their surface, and this differentiation has enabled the development of novel therapeutic agents through highly specific targeting [31,32].

**Figure 1:**
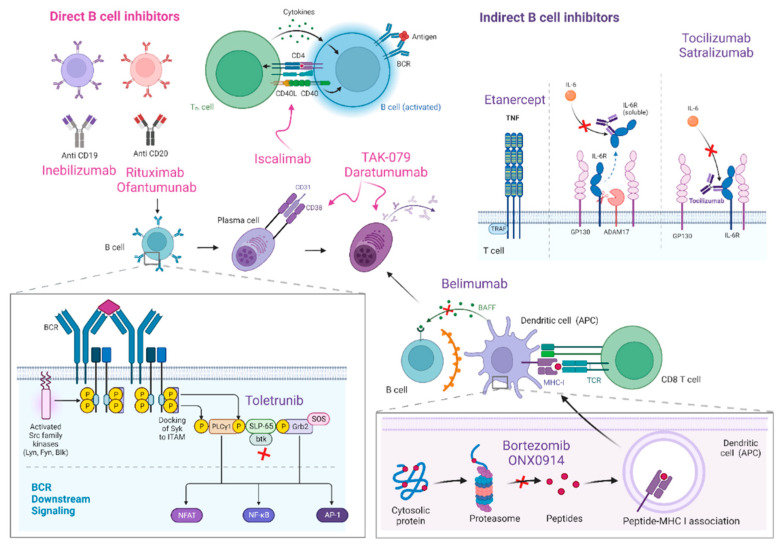
B-cell inhibitors and their main mechanisms of action are represented in this figure. Direct B cell inhibitors include monoclonal antibodies against CD19 (inebilizumab) and CD20 (rituximab and ofantumumab) B cell surface proteins, as well as iscalimab, a monoclonal anti-body against CD145-CD40. Drugs targeting plasma cells comprise proteasome inhibitors (borte-zomib and ONX0914) and anti-CD38 medications (mezagitamab or TAK-079 and daratumumab). Indirect B cell inhibitors are drugs designed to block IL-6 (tocilizumab and satralizumab), TNF (etanercept), BAFF (belimumab) or BTK (toletrunib).

Drugs acting selectively against B cells have several uses in rheumatological diseases, hematological malignancies, and even other autoimmune neurological disorders such as multiple sclerosis. In recent years, they have also been used as novel key therapies in MG [33,34] (Figure 1).

#### 3.1.1. Direct B-Cell Inhibitors

1. Rituximab (RTX)

Rituximab is a murine-human chimeric anti-CD20 glycoprotein monoclonal antibody. It has fragmented antigen-binding (Fab) region domains that target CD20-expressing B cells, but spare B cells in the bone marrow and lymph nodes, as well as stem cells, pro-B cells, and long-lived plasma cells and plasmablasts [35,36] (Figure 1).

The use of rituximab in MG has increased exponentially in the last 10–15 years. However, the level of evidence for this drug in MG is low based on retrospective and prospective observational studies. The efficacy outcome in these studies (acquiring minimal manifestation status or better) was achieved in around 50% of the rituximab-treated patients, while considering different population inclusion criteria [37,38,39,40,41,42]. Even at low doses, rituximab seems to be effective in some MG patients [43,44,45].

Rituximab has proven to be more beneficial in MuSK-MG. A blinded, multicenter, prospective review showed that more than 55% of MuSK-MG patients treated with rituximab reached the primary outcome measure compared with 16% of controls [46]. In addition, some studies showed a significant reduction in MuSK-IgG4 antibody levels in rituximab-treated patients in clinical remission with sustained improvement [47]. Other authors have compared the efficacy of different rituximab regimens [48], hypothesizing that the superior effect of a specific treatment regimen could be explained by the reduction in short-lived plasma cells (which are considered the primary source of MuSK antibody production), whereas long-lived plasma cells (not expressing CD20) are the major AChR-ab producers [29,49]. However, the most recent international consensus guidelines on the management of MG state that the role of rituximab in refractory MG is unclear [2].

Despite the scarce evidence from more prospective or controlled studies, the abundance of real-life data indicates that the early administration of rituximab is recommended in patients with MuSK antibodies. However, the most recent international consensus guidelines on the management of MG are not clear on the administration of this agent in patients with AChR-abs [2].

The only phase 2 clinical trial comparing rituximab with a placebo as an add-on treatment in AChR-MG patients recently reported disappointing results, namely, no significant differences in disease severity or corticosteroid-sparing effect over placebo [50]. More recently, results from a randomized trial using a single-dose of rituximab in AChR-positive gMG patients with a Quantitative MG (QMG) score >6 and a short disease course (<12 months) showed benefits in clinical outcomes and a reduction in the use of rescue medication [51]. Further randomized clinical trials with rituximab are needed, particularly in refractory MG patients, in whom therapeutic options are limited.

Rituximab is generally well-tolerated, with few severe adverse effects, mainly hypogammaglobulinemia or, very rarely, progressive multifocal leukoencephalopathy [52,53]. A specific rituximab dosage was not clearly outlined in the last consensus guidelines [2]. Different therapeutic regimens are used; however, the most common schemes were recently revealed in a literature review, namely Rituximab 375 mg/m^2^ body surface in a 4-week cycle with weekly infusions or two infusions of 500–1000 mg at days 1 and 15 [54].

2. Other B-cell inhibitors

No evidence is available for new-generation anti-CD20 agents such as ocrelizumab, obinutuzumab, ublituximab, and veltuzumab in the treatment of MG, whereas new anti-CD19 treatments are being proposed [55,56]. A case of a patient with refractory AChR-MG responding to ofatumumab has been reported [57].

Anti-CD19 drugs could have some advantages over anti-CD20 agents, as the CD19 marker is expressed much earlier than CD20 in the B-cell maturation process and might act synergistically with anti-CD20 agents [55].

Inebilizumab is a humanized, afucosylated IgG1 kappa monoclonal antibody that depletes CD19-expressing B cells through antibody-dependent cell-mediated cytotoxicity mechanisms [58]. It was approved for neuromyelitis optical spectrum disorder (NMOSD) after a successful phase 2/3 trial [59]. A phase 3 study is currently underway in seropositive gMG [60].

Iscalimab is a fully human, Fc-silenced, IgG1 mAb that blocks the CD40 signaling pathway by binding with its ligand (CD154), which is expressed in activated T cells. This union enhances the immune response by promoting proinflammatory cytokine secretion and dendritic cell activation [61]. A multicenter, randomized, double-blind, placebo-controlled phase II clinical trial in seropositive gMG has been completed. The as yet unpublished results indicate that the outcome measure of significant improvement in MG scores was not reached, although there were no safety concerns [62] (Figure 1).

3. Drugs targeting plasma cells

Targeting long-lived memory plasma cells may be an attractive therapeutic approach in patients with antibody-mediated refractory diseases such as MG. The surface markers of these cells differ from those of B cells and may therefore be resistant to the therapies mentioned above.

- Proteasome inhibitors

In cells characterized by highly active immunoglobulin synthesis, such as plasma cells, the inhibition of the proteasome function leads to an accumulation of misfolded proteins and to apoptosis. This therapeutic strategy has proven to be effective in B-cell neoplasms such as multiple myeloma [63]. Bortezomib is the most studied proteasome inhibitor in MG. In animal models of experimental autoimmune MG (EAMG), bortezomib showed positive effects, including a reduction in AChR-ab levels [64].

A non-randomized clinical trial with bortezomib in patients with antibody-mediated autoimmune diseases, including MG, was terminated owing to recruitment difficulties [65], thus necessitating further studies on the role of bortezomib in MG. The adverse effects of this drug include frequent neurotoxicity and the consequent disabling peripheral neuropathy [66]. More selective proteasome inhibitors could overcome these barriers, since they are more effective and safe in MG. One example of new-generation proteasome inhibitors is ONX0914, which has proven to be successful in EAMG models [67] (Figure 1).

b. Biologic drugs targeting plasma cells

Drugs targeting the surface proteins of plasma cells constitute yet another therapeutic option. On this basis, various monoclonal antibodies against the glycoprotein CD38 expressed in Ig-secreting plasma cells and thymocytes [68,69] are now being studied for MG. Mezagitamab was recently evaluated in a randomized phase 2 trial, although the results have yet to be published [70]. Another anti-CD38 antibody, daratumumab, has been evaluated in a retrospective, single-centre case series of seven patients with autoantibody-driven neurological autoimmune diseases, including one patient with MG. The preliminary results are promising [71] (Figure 1).

#### 3.1.2. Indirect B-Cell Inhibitors

1. Cytokines, interleukins, and other immune mediators

T-cell dysfunction and an altered balance of pro-and anti-inflammatory cytokines and interleukins are common features in the pathogenesis of numerous autoimmune diseases (AIDs).

One of the most studied mediators within this therapeutic group is the IL-6 pathway. IL-6 is involved in signaling cascades promoting B-cell differentiation and the switch from Treg to Th17 cells. As previously noted, the imbalance between Treg and Th17 appears to play a pathogenic role in EAMG models, with a decrease in autoantibody titers before IL-6 blockade [72].

Tocilizumab is a well-known anti-IL-6 monoclonal antibody that has proven effective in cases of severe refractory MG [73]. One clinical scenario in which tocilizumab may play a special role is that of myasthenic crisis in the context of COVID-19, where there are isolated reports of its effectiveness [74]. A phase 2 clinical trial with tocilizumab in patients with positive AChR-ab gMG is planned to start soon [75].

Satralizumab is a monoclonal antibody that blocks the IL-6 receptor. A phase 3 clinical trial is currently enrolling patients to evaluate the efficacy and safety of satralizumab in gMG [76]. The modulation of other cytokines by monoclonal antibodies, such as secukinumab (anti-IL-17A), may prove to be a promising option [56].

Given the role of the proinflammatory cytokine tumor necrosis factor (TNF) in the pathogenesis of many AIDs, anti-TNF agents are widely used. A prospective pilot clinical trial with etanercept in corticosteroid-dependent MG showed the drug’s efficacy in patients with low levels of interferon-gamma and IL-6. Conversely, patients with elevated levels of these cytokines experienced clinical deterioration, and there are individual reports of the worsening of MG symptoms with these drugs [77,78]. Considering the uncertain risk of other immune-mediated phenomena with the long-term use of anti-TNF agents, further studies are necessary to determine their benefit in MG (Figure 1).

2. B-cell-activating factor

B-cell-activating factor (BAFF), or B-lymphocyte stimulator (BLyS), is a member of the TNF superfamily that promotes B-cell survival and co-stimulates its functions [79]. Its role as a potential therapeutic candidate has been suggested in studies showing elevated levels in the serum of patients with MG [80]. However, a randomized phase 2 trial comparing belimumab with placebo in patients with anti-AChR-ab+ gMG revealed no statistically significant differences for the clinical endpoints (MG-Activities of Daily Living [ADL] and QMG score) at week 24 [81]. Despite these negative results, the conclusions of the study could be affected by its limitations, one of which was its heterogeneous population, including patients with mild symptoms on standard therapy (Table 1, Figure 1).

3. Bruton’s tyrosine kinase

Bruton’s tyrosine kinase (BTK) is a critical element in the B-cell signaling downstream, as are other sensors related to innate immunity, such as Toll-like receptors [82]. The inhibition of BTK is associated with reduced activation, maturation, and antibody production and modulates the activation of other hematopoietic cells [83]. Tolebrutinib is an oral BTK inhibitor that is being developed for the treatment of MG and multiple sclerosis in a phase 2b clinical trial, where it showed an acceptable safety and tolerability profile [84]. A phase 3 clinical trial was recently initiated to assess the efficacy of tolebrutinib in gMG [85]. However, recruitment has been momentarily halted based on the recommendations of the independent data monitoring committee owing to a limited number of cases of drug-induced liver injury (Table 1, Figure 1).

### 3.2. Complement Inhibitors

Complement activation is one of the most significant pathogenic mechanisms of AChR-MG [86,87], and its role in the pathogenesis of MG is supported by histopathological findings of C3 and C9 deposits at the NMJ [86,88], as well as an increased in vitro uptake of complement C3b in serum [89]. In addition, increased complement consumption during MG exacerbations resulting from changes in the serum levels of various complement proteins have been reported in MG patients [90]. Likewise, EAMG models have demonstrated that complement deposition at the NMJ results in the destruction of the postsynaptic endplate, with similar findings in human muscle samples [91]. These results reinforce the role of complement activation as the key element for the development of MG in animal models [92]. Therefore, it is reasonable to develop complement inhibitors as new targeted therapies for MG.

As mentioned above, MuSK-MG antibodies are mainly a subclass IgG4, in contrast to AChR antibodies, which are subclasses of IgG1 to 3. Therefore, in these cases, activation of the complement pathway is not involved in pathophysiology [20], and, in theory, a response to complement inhibitors would not be expected in MuSK-MG patients.

1. Eculizumab

**Figure 2:**
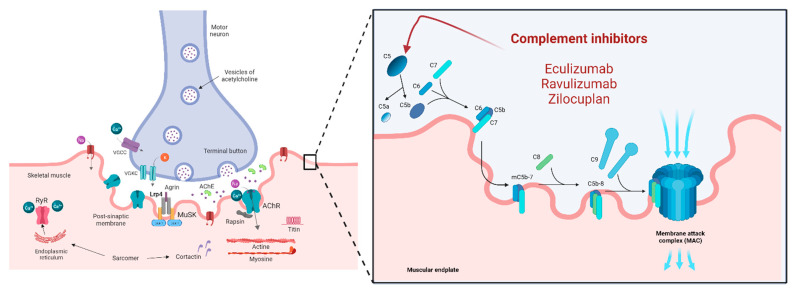
Complement inhibitors and their mechanisms of action are represented in this figure. At the muscular endplate, the complement system activates the membrane attack complex (MAC) as its last step, contributing to the destruction of the architecture of the muscular surface in MG. Eculizumab, Ravulizumab and Zilocuplan inhibit the complement system by blocking C5 action.

Eculizumab (Solaris) is a humanized monoclonal antibody that inhibits the cleavage of complement protein C5 into its terminal active components, C5a and C5b (Figure 2). It is the first complement inhibitor approved for the treatment of refractory anti-AChR+ gMG [2]. However, given its high cost, its use is highly restricted in some countries.

In the phase 3 (REGAIN) trial, the primary endpoint (change in the MG-ADL score from baseline to week 26) did not differ significantly between the eculizumab and placebo arms (*p* = 0.0698), possibly owing to the statistical analysis method, namely, worst-rank analysis [93]. However, all secondary endpoints showed a significant benefit from eculizumab [94]. One of the most dangerous complications expected with complement inhibitor therapies is infection with encapsulated bacteria, mainly Neisseria meningitides, although all patients in the trial were vaccinated and none developed a meningococcal infection.

After completion of the REGAIN trial, patients could enter the open-label extension phase [95]. At the end of the study, it was possible to reduce the mean daily doses of conventional immunosuppressive therapy from baseline to the last assessment (by 60.8% in the case of prednisone, by 89.1% in the case of azathioprine, and by 56.0% in the case of mycophenolate mofetil) [96]. There was one case of non-fatal meningitis during the extension phase.

Future studies should evaluate the required duration of eculizumab to maintain treatment goals and efficacy in other MG populations, such as patients with thymoma and patients with seronegative MG [2].

It is necessary to administer anti-meningococcal vaccination (meningococcal conjugate Men ACWY and serogroup B or MenB) before starting eculizumab [2].

2. Ravulizumab

Ravulizumab (Ultomiris^®^) has the same mechanism of action as eculizumab, i.e., it binds to C5, thus preventing the generation of the complement activation products C5a and C5b-9 [97] (Figure 2). Ravulizumab was developed with the intention of extending the intravenous dosing schedule of eculizumab every two weeks. This molecule is obtained through selective modifications of eculizumab to abolish target-mediated drug disposition and to increase recycling efficiency via FcRn in the immunoglobulin pathway, with the aim of extending maintenance dosing to an interval of 8 weeks [98].

Ravulizumab is currently approved in the U.S., Europe, and other regions for the treatment of atypical hemolytic uremic syndrome and paroxysmal nocturnal haemoglobinuria. A phase 3 trial evaluating the safety and efficacy of ravulizumab in patients with gMG was recently completed with positive results in MG-ADL and QMG scores in patients compared to placebo from the baseline visit to week 26 [99] (Table 1).

3. Zilucoplan

Zilucoplan prevents the cleavage of C5 into complement components C5a and C5b and blocks the binding of C5b to complement component C6 [100] (Figure 2). Compared to eculizumab, it has the advantage of self-administered subcutaneous dosing.

A phase 2 trial evaluated the clinical effects of zilucoplan in patients with moderate-to-severe gMG, obtaining favorable results, especially using the 0.3 mg/kg daily dose [100].

A phase 3 trial with a daily dose of 0.3-mg/kg in adults with gMG was recently completed [101]. However, as in the case of ravulizumab, no data have been published, and only the press release from the pharmaceutical company is currently available. The trial met the primary endpoint (MG-ADL score) and all key secondary endpoints (QMG score, MGC, and MG-QoL15r) [102] (Table 1).

### 3.3. FcRn Inhibitors

**Figure 3:**
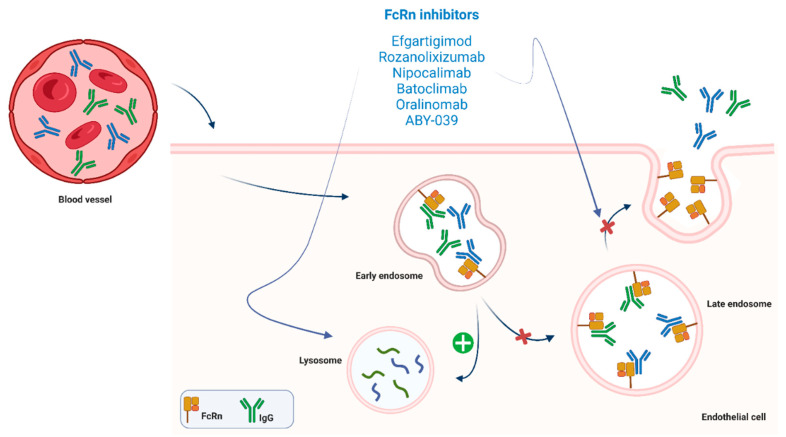
FcRn-targeted drugs exert is mechanism of action by inhibiting FcRn functions. These drugs favor lisosomal pathways activation and inhibit endosomal recycling of IgG. Their actions result in a drastic diminution of pathogenic IgG blood levels.

IgG is conventionally recycled via the endosomal–lysosomal system in the cytoplasm by endothelial or blood cells (e.g., monocytes). The FcRn (which belongs to the Fc-gamma [Fc-ɣ] receptor superfamily) is expressed by these endothelial cells and binds IgG at the acidified early endosome. IgG-FcRn binding protects IgG from lysosomal degradation and favors its recycling. IgG then becomes free again at a physiological pH to return to the bloodstream. FcRn is also responsible for albumin degradation, although its binding site differs from the IgG site and they are not competitive [103,104] (Figure 3).

Anti-AChR auto-antibodies (IgG) play a key role in the pathogenesis of MG. Drugs targeting FcRn are monoclonal antibodies designed to counteract the humoral response in MG and other autoimmune diseases by inhibiting FcRn functions. FcRn inhibitors are able to lower IgG levels drastically, by reducing the lysosomal recycling of IgG and, as a consequence, enhancing the elimination of pathogenic IgG [104,105,106,107]. As a result, the effect of FcRn inhibitors on antibody levels’ reduction resembles that of plasmapheresis (PLEX), except that these novel drugs are much better tolerated and have less severe complications than PLEX. 

#### 3.3.1. Efgartigimod

Efgartigimod is an FcRn inhibitor consisting of engineered human monoclonal antibodies against the FcRn fragment of the IgG1. Its efficacy and safety as a specific, potent agent causing an average sustained reduction of 75% in serum IgG levels was initially proven in a first-in-human study [108] (Figure 3); subsequently, in a phase 2 trial, Efgartigimod achieved a rapid and long-lasting clinical improvement that correlated with a fast and durable decrease in total IgG and AChR-ab levels [109].

The results of the phase III (ADAPT) study for efgartigimod were recently published [110]. This multicenter, double-blind, randomized trial included gMG patients from 56 centers worldwide.

Efgartigimod caused no serious safety concerns, and its efficacy results were excellent. Most of the patients in the efgartigimod group were MG-ADL responders after the first treatment cycle (4 weeks), compared to patients in the placebo group [110].

A new phase 3, randomized, open-label study comparing the pharmacodynamics, pharmacokinetics, efficacy, safety, tolerability, and immunogenicity of subcutaneous and intravenous efgartigimod in gMG was completed in 2021. Results from this trial have not yet been published [111], although an open-label trial evaluating the long-term safety and tolerability of subcutaneous efgartigimod is ongoing [112] (Table 1).

The intravenous presentation of efgartigimod (Vyvgart™) received U.S. Food and Drug Administration approval for the treatment of adult, AChR-ab–positive patients with generalized myasthenia gravis (gMG) in December 2021 [113] and European Medicines Agency approval in September 2022 [114].

Efgartigimod (20 mL, 400 mg, 20 mg/mL vials) is recommended to be administered intravenously. The recommended dosage according to the EMA product information brochure is 10 mg/kg in a 1-h infusion and in once weekly cycles for 4 weeks. A patient’s clinical follow-up is crucial to decide the periodicity of the subsequent treatment cycles. According to the previous evidence of efgartigimod, subsequent cycles must be administered at least 7 weeks from the initial infusion of the first cycle, but not earlier.

The more common side effects of efgartigimod are upper respiratory tract infections (>1/10) and urinary infections, bronchitis, myalgia and procedural headache (≥1/100 to <1/10). 

An expanded access program for the administration of intravenous efgartigimod is now available to patients with AChR-positive gMG [115].

#### 3.3.2. Rozanolixizumab

Rozanolixizumab is another FcRn antagonist (Figure 3); it is based on humanized, high affinity, human IgG4 anti-FcRn monoclonal antibodies [116].

Results from a phase 2 clinical trial were also published in 2021 (MG003) [117]. In the first study period, patients were randomized (1:1) to receive once weekly 7 mg/kg subcutaneous rozanolixizumab or placebo doses from day 1 to 29 in two periods. Patients were then re-randomized to rozanolixizumab 7 mg/kg or 4 mg/kg once weekly for 3 weeks and observed until day 99. The primary endpoint was not attained, as changes in QMG from baseline were not statistically significant between groups; no serious safety concerns were reported. 

Phase 3 trial results for rozanolixizumab (MG004) have not yet been published, although a recent press release from the company announces positive findings [102]. An active open-label study (MG007) to prove its long-term safety is estimated to be completed in October 2023 [118] (Table 1).

#### 3.3.3. Nipocalimab

The FcRn inhibitor nipocalimab (M281) is a fully human alpha-deglycosylated IgG1 anti-FcRn monoclonal antibody (Figure 3). This molecule significantly reduced IgG levels in a dose-dependent and sustained way in the phase I study. M281 acts selectively by binding, saturating, and blocking the IgG binding site on the endogenous FcRn [119], and its transplacental transfer rate is extremely low [93].

A phase 2 trial (VIVACITY-MG) revealed no tolerance or safety concerns when assaying intravenous ascending doses of nipocalimab [120]. The phase 3 nipocalimab trial, which is designed to assess both efficacy and safety in adult gMG, is actively recruiting patients. This trial and the following open-label extension study are expected to finish by 2026 [121]. 

An expanded access program for nipocalimab has been available for adult patients with warm autoimmune haemolytic anaemia from February 2022 [122].

#### 3.3.4. Batoclimab

Batoclimab, also known as RVT-1401 or HL161, is a human recombinant anti-FcRn monoclonal antibody [123] currently under evaluation for the treatment of gMG (Figure 3). 

It is administered subcutaneously and has already proven to be effective and safe in a double-blind, placebo-controlled phase 2 study in gMG patients [124]. 

Patients are still being recruited in a phase 3 trial that aims to assess the efficacy and safety of batoclimab as induction and maintenance treatment for adults with gMG. The study comprises 2 periods: in the first, patients are randomized 1:1:1 to two different doses of batoclimab or placebo, and, in the second, patients treated with batoclimab initially are to be re-randomized to once- or twice-weekly low-dose batoclimab or placebo. Patients who respond to batoclimab will be considered candidates for an open-label extension study [125] (Table 1).

#### 3.3.5. Other FcRn Antagonists and Related Drugs

Orilanomab (SYNT001 or ALX1830) is a human recombinant monoclonal IgG4 antibody against FcRn [126] (Figure 3). A phase 1 study reported pharmacokinetics and pharmacodynamics results in humans after accurate characterization of the molecule and its effects on animal models [126]. Two phase 1B/2 trial were developed and completed in 2019 in patients with warm autoimmune haemolytic anaemia [127] and chronic pemphigus. Efficacy and tolerability outcomes were positive, with rapid and significant reduction of IgG and IgG immunocomplexes [128].

ABY-039 is a bivalent antibody-mimetic (a 18KDa peptide) that targets the FcRn. A phase 1 trial had to be prematurely interrupted owing to tolerability concerns [129]. This molecule also theoretically proved to be a potent agent with the ability to lower plasma IgG levels and had a very long half-life in vivo [123,130,131].

## 4. Discussion and Conclusions

During the last decade, treatment of MG has undergone considerable changes, mainly owing to the advent of multiple new biological drugs to improve disease outcomes. Clinical trials have reported positive results for some of these drugs, which have specific immunological targets, and other studies are ongoing. 

Potential FcRn-related drugs for MG, such as molecules targeting FcɣR or drugs based on recombinant Fc multimers, are currently being investigated in pre-clinical, phase 1, and phase 2 studies or in other AIDs [107,123]. In addition, CAR-T or CAAR-T cell therapies, which are based on modified T cells expressing chimeric antigen receptors, have also been pre-clinically assayed or are currently being trialled in other AIDs [93]. 

It is important to consider the global impact of this changing paradigm in MG and in other, similar antibody-mediated AIDs, with emphasis on the refinement of therapies that target B cells and complement and the promising results of efgartigimod. Therefore, this new era in disease treatment and management is leading experts to progressively introduce immunological targeted therapies in a more personalized patient-centred model. This debate is crucial for patients with a refractory phenotype, usually with substantial comorbidities, and whose quality of life is often devastated by MG. 

Other authors have postulated that some concrete circulating miRNAs (e.g., miR-150 or miR-21-5p) could act as MG biomarkers, by helping experts to stratify MG patients ac-cording to their disease onset or their serological profile. Based on the upregulation of these biomarkers and their influence in MG pathophysiology, they hypothesized that these circulating miRNAs may have a role in the design of personalized treatment schemes [132].

Despite the lack of concern about the elevated cost of these novel drugs (as with eculizumab), little is known about their future market prices and their real cost-effectiveness.

**Table 1:** Main targeted drugs in myasthenia gravis.

Drug	Target	Product	Type of Study ^†^	MG Population	Study Code	Results ^ђ^	Reference ^φ^
B-cell inhibitors							
Direct B-cell inhibitors							
Rituximab	CD20	Murine-human chimeric IgG1k mAb	Phase 2 Phase 3 Non-randomized observational	AChR-MG AChR and seronegative MG MuSK-MG and AChR-MG	NCT02110706 NCT02950155	Controversial in AChR-MG Positive in AChR-MG, single dose of 500 mg rituximab Positive in MuSK-MG	Nowak et al., 2022 [46] Piehl et al., 2022 [47] Beecher et al., 2018: Topakian et al., 2019; Dos Santos et al., 2020; Brauner et al., 2020; Choi et al. 2019; Lu et al., 2020; Li et al., 2021; Hehir et al. 2017; Cortés-Vicente et al. 2018 [34,35,36,37,38,39,40,41,42,44].
Ofatumumab	CD20	Fully human IgG1k mAb	Non-randomized, single case report	AChR-MG, refractory, previously treated with rituximab	NA	Not applicable	Waters et al., 2019 [52]
Inebilizumab	CD19	Humanized, afucosylated IgG1k mAb	Phase 3	AChR-gMG	NCT04524273	Pending	ct.gov [55]
Iscalimab (CFZ533)	CD145-CD40	Fully human, Fc-silenced, IgG1 mAb	Phase 3	AChR-gMG	NCT02565576	Pending	ct.gov [57]
**Drugs targeting plasma cells**							
**Proteasome inhibitors**							
Bortezomib	Proteasome		Non-randomized clinical trial	Antibody-mediated AIDs, including MG	NA	Not completed, neurotoxicity	Kohler et al., 2019 [60]
ONX0914	Proteasome		Pre-clinical	EAMG models only	NA	Positive	Liu et al., 2017 [62]
**Biologic drugs against plasma-cells**							
Mezagitamab (TAK-079)	CD38		Phase 2	AChR-gMG MuSK-MG	NCT04159805/EudraCT:2019-003383-47	Pending	ct.gov [65]
Daratumumab	CD38	Human IgG1k mAb	Non-randomized, retrospective, single-centre study	n = 7, 1 MG	NA	Positive	Scheibe et al., 2022 [66]
**Indirect B-cell inhibitors**							
Tocilizumab	IL-6		Phase 2	AChR-gMG	NCT05067348	Pending start	ct.gov [70]
Satralizumab	IL-6		Phase 3	AChR-gMG	NCT04963270	Pending, recruiting	ct.gov [71]
Etanercept	TNF		Non-randomized, prospective (pilot)	AChR-gMG, corticosteroid-dependent	NA	Controversial, toxicity	Pelechas et al., 2020 [72]
Belimumab	BAFF		Phase 2	AChR- gMG	NCT01480596	Negative	Hewett et al. 2018 [76]
Tolebrutinib	BTK		Phase 3	AChR- gMG	NCT05132569/EudraCT: 2021-003898	Pending, halted recruitment	ct.gov [80]
**Complement inhibitors**							
Eculizumab	C5		Phase 3 and OLE	AChR-gMG, refractory	NCT01997229 and NCT02301624	Approved for the treatment of refractory anti-AChR+ gMG (Solaris^®^) Phase 3: 900 mg intravenous on days 1 and at weeks 1, 2, and 3, followed by 1200 mg at week 4 and 1200 mg every 2 weeks as a maintenance dose. Primary endpoint not met (change in MG-ADL from baseline to week 26). Significant differences in secondary endpoints (changes in QMG, MG Composite, and MG-QOL15 scores). OLE: all patients received eculizumab maintenance therapy (1200 mg every 2 weeks), 90% patients improved, 60% remission	Howard et al., 2017 [89]
Ravulizumab	C5		Phase 3	AChR-gMG	NCT03920293	Positive Patients receive a loading dose on day 1, followed by maintenance doses on day 15 and every 8 weeks thereafter. Loading dose: 40 to <60 kg: 2400 mg IV; 60 to <100 kg: 2700 mg IV; ≥100 kg: 3000 mg IV Maintenance IV dose: 40 to <60 kg: 3000 mg IV; Q8W; 60 to <100 kg: 3300 mg IV Q8W; ≥100 kg: 3600 mg IV Q8W	Tuan et al., 2022 [94]
Zilucoplan	C5		Phase 3	AChR-gMG	NCT04115293	Pending phase 3 results, preliminary positive (press release)	ct.gov [96]
**FcRn inhibitors**							
Efgartigimod	FcRn	Human FcRn mAb	Phase 3	AChR-gMG MuSK-MG	NCT04735432	Approved in the US and Europe for the treatment anti-AChR+ gMG (Vyvgart^®^). 68% MG-ADL responders and 34% MG-ADL 0-1 (minimal symptom) by the end of first in the first treatment cycle. Higher MG-ADL early-responder (2 weeks) proportion vs placebo group. Improvement in QMG, MGC, MG-QoL15 at 7 weeks after first infusion.	Howard et al., 2021 [105]
Rozanolixizumab	FcRn	Humanized, high-affinity, human IgG4 anti-FcRn mAb	Phase 3	AChR-gMG MuSK-MG	NCT04124965	Pending publication phase 3, positive results (press release) Primary and all secondary endpoints with statistical significance and no safety or tolerance concerns	Bril et al., 2021 [112]
Nipocalimab (M281)	FcRn	Fully human alpha-deglycosylated IgG1 anti-FcRn mAb	Phase 3	AChR-gMG MuSK-MG	NCT04951622	Positive phase 2, pending phase 3 results	ct.gov [117]
Batoclimab (RVT-1401 or HL161)	FcRn	Human recombinant anti-FcRn mAb	Phase 3	AChR-gMG MuSK-MG	NCT05403541	Pending phase 3, recruiting	ct.gov [121]
Oralinomab (SYNT001 or ALX1830)	FcRn	Human recombinant IgG4 anti-FcRn mAb	No trials in MG	NA	Phase 1/2 in wAIHA (NCT03075878) and chronic pemphigus (NCT03075904)	Not applicable to MG	ct.gov [123]; Werth et al., 2021 [124]

Main targeted drugs in myasthenia gravis, their composition, immunological target, and mechanism of action. The table also shows clinical evidence based on previous studies or randomized clinical trials, as well as the results of these studies. ^†^: type of study includes the latest clinical trial phase (active or completed); ^ђ^: dosage has only been included for the approved medications; ^Φ^: studies without available publications have been cited using ct.gov (https://clinicaltrials.gov (accessed on 31 August 2022)) including registered NCT code. Abbreviations: mAb—monoclonal antibody; gMG—generalized myasthenia gravis; AChR-MG—MG with positive antibodies against acetylcholine receptor; MuSK-MG—MG with positive antibodies against MuSK protein; AIDs—autoimmune diseases; EAMG—experimental autoimmune myasthenia gravis; OLE- open-label extension study; NA: not available; Q8W: every 8 weeks; wAIHA: warm autoimmune hemolytic anemia.

## Data Availability

Not applicable.

## Abbreviations

MG—myasthenia gravis; NMJ—neuromuscular junction; mAb—monoclonal antibody; Treg—regulatory T cells; AChR—acetylcholine receptor; MuSK—muscle-specific kinase; LrP4—low-density lipoprotein receptor type 4;gMG—generalized myasthenia gravis; Fab—fragment antigen-binding; AChR-MG—MG with positiveantibodies against acetylcholine receptor; MuSK-MG—MG with positive antibodies against MuSK protein; AIDs—autoimmune diseases; EAMG—experimental autoimmune myasthenia gravis; TNF—tumour necrosis factor; BAFF—B-cell-activating factor; BlyS—B-lymphocyte stimulator; BTK: Bruton’s tyrosine kinase; MG-ADL- MG activities of daily live score; QMG—quantitative myasthenia gravis score; MGC—MG composite score; MG-QoL15r—MG quality of life (15 items) revised score; FcRn—neonatal fragment crystallizable receptor; OLE—open-label extension study; wAIHA: warm autoimmune haemolytic anaemia; CAR-T—chimeric antigen receptors therapies.
